# Antibiotic consumption and antimicrobial resistance in the SARS-CoV-2 pandemic: A single-center experience

**DOI:** 10.3389/fphar.2023.1067973

**Published:** 2023-03-15

**Authors:** Dragana Sokolović, Dragana Drakul, Vesna Vujić‐Aleksić, Bojan Joksimović, Siniša Marić, Lana Nežić

**Affiliations:** ^1^ Department of Pharmacology, Faculty of Medicine Foča, University of East Sarajevo, Foča, Bosnia and Herzegovina; ^2^ Centre for Biomedical Research, Faculty of Medicine Foča, University of East Sarajevo, Foča, Bosnia and Herzegovina; ^3^ The Republic of Srpska Agency for Certification, Accreditation and Quality Improvement in Health Care, Banja Luka, Bosnia and Herzegovina; ^4^ Department of Pharmacology, Toxicology and Clinical Pharmacology, Faculty of Medicine, University of Banja Luka, Banja Luka, Bosnia and Herzegovina; ^5^ Department of preclinical subjects (Pathophisiology), Faculty of Medicine Foča, University of East Sarajevo, Foča, Bosnia and Herzegovina; ^6^ Department of Surgery, Hospital “Saint Apostol Luka”, Doboj, Bosnia and Herzegovina

**Keywords:** antimicrobial resistance, antibiotic consumption, reserve antibiotics, multiresistant bacteria, COVID-19

## Abstract

**Introduction:** Antimicrobial resistance and the rapid spread of multiresistant bacteria represent one of the main public health problem in limited resources countries. This issue is significantly worsening since the COVID-19 pandemic due to the unreasonably increased antibiotics prescription to patients with confirmed SARS-CoV-2 infection. The aim of this study was to examine whether COVID-19 pandemic (2020, 2021) was associated with increased antibiotic consumption in inpatient and outpatient settings in the middle size urban region (Republic of Srpska/Bosnia and Herzegovina) in comparison to period before the pandemic (2019). Additionally, we aimed to determine antimicrobial resistance and the presence of multiresistant bacteria in the regional hospital (“Saint Apostol Luka” Hospital Doboj) in 2021.

**Methodology:** The consumption of antibiotics in inpatient was calculated as Defined Daily Dose per one hundred of patient-days. The consumption of antibiotics in outpatient was calculated as Defined Daily Dose per thousand inhabitants per day. Resistance of bacteria to antibiotics is expressed as a rates and density for each observed antibiotic. The rate of resistance was calculated as a percentage in relation to the total number of isolates of individual bacteria. The density of resistance of isolated bacteria against a specific antibiotic was expressed as the number of resistant pathogens/1000 patient days.

**Results:** Antibiotic consumption in hospital setting registered during 2019, 2020 and 2021 was as follows: carbapenems (meropenem: 0.28; 1.91; 2.33 DDD/100 patient-days, respectively), glycopeptides (vancomycin: 0.14; 1.09, 1.54 DDD/100 patient-days, respectively), cephalosporins (ceftriaxone: 6.69; 14.7; 14.0 DDD/100 patient-days, respectively) and polymyxins (colistin: 0.04; 0.25; 0.35 DDD/100 bed-days, respectively). Consumption of azithromycin increased drastically in 2020, and dropped significantly in 2021 (0.48; 5.61; 0.93 DDD/100 patient-days). In outpatient setting, an increase in the consumption of oral forms of azithromycin, levofloxacin and cefixime, as well as parenteral forms of amoxicillin-clavulanic acid, ciprofloxacin and ceftriaxone, was recorded. In 2021, antimicrobial resistance to reserve antibiotics in hospital setting was as follows: Acinetobacter baumanii to meropenem 66.0%, Klebsiella spp to cefotaxime 67.14%, Pseudomonas to meropenem 25.7%.

**Conclusion:** Recent COVID-19 pandemic was associated with increased antibiotic consumption in inpatient and outpatient settings, with characteristic change of pattern of azithromycin consumption. Also, high levels of antimicrobial resistance to reserve antibiotics were registered in hospital setting with low prevalence of identified pathogen-directed antimicrobial prescription. Strategies toward combat antimicrobial resistance in the Doboj region are urgently needed.

## 1 Introduction

After the isolation of a new strain of the virus called SARS-CoV-2 in Wuhan, China, it was found that the new respiratory virus caused the illness of a large number of people with huge differences in the severity of the clinical picture and the course of the disease, from mild cases without significant symptoms to the most severe cases of pneumonia with the need for artificial ventilation (Zhu et al., 2019). A large number of patients, overloading of health systems, ignorance of the disease itself, lack of effective antiviral drugs, and fear of the development of bacterial co-infections have led to an increased prescription of antibiotics. In the months that followed, although knowledge about COVID-19 expanded significantly, the overlap of clinical and radiological features of COVID-19 with a bacterial infection of the respiratory tract helped to maintain the trend of increased and unnecessary antibiotic prescription. Numerous studies have reported an unjustified increase in antibiotic use during the COVID-19 pandemic, potentially accompanied by the rapid development and evolution of antibiotic resistance (Rawson et al., 2020; Grau et al., 2021a; Martinez-Guerra et al., 2021; Sokolović et al., 2022). Studies conducted so far show that the use of antibiotics in patients with COVID-19 greatly exceeds the number of proven bacterial superinfections (Rawson et al., 2020). The most common causative agents of bacterial superinfections in patients with COVID-19 are *Acinetobacter baumannii*, *Mycoplasma pneumoniae*, *Pseudomonas aeruginosa*, and *Haemophilus influenzae*, and they differ from the causative agents in patients co-infected with other respiratory viruses. *Streptococcus pneumoniae*, *Staphylococcus aureus*, and *Streptococcus pyogenes* are most often present in sputum and bronchoalveolar aspirate of patients with influenza (Lucien et al., 2021). The emergence of vaccines and the development of natural and artificial immunity to SARS-CoV-2 reduced the initial fear and we learned to live with this virus. On the other hand, the development of antimicrobial resistance (AMR) represents a global threat to public health systems worldwide. As a basic and necessary measure to bring AMR under control, the WHO recommended the establishment of a system to monitor the consumption of antimicrobial agents (World Health Organization, 2020). Surveillance and optimization of antibiotic use is necessary to control AMR and is one of the global action plans in the fight against AMR by the WHO (World Health Organization, 2015; World Health Organization, 2020).The WHO developed the AWaRe (Access, Watch, and Reserve) classification of antibiotics as a tool to combat AMR (World Health Organization, 2019a; Rashid et al., 2022). The Access group encompasses 48 wide-spectrum antibiotics with a lower resistance potential than antibiotics from other groups. The Watch group includes antibiotics with a higher resistance potential that should be carefully monitored within the antibiotic stewardship program (ASP). Antibiotics of Access and Watch groups should be the first or second choice for empiric treatments of specified infectious syndromes. The Reserve group includes antibiotics that should be accessible but are reserved for the treatment of confirmed or suspected infections caused by multidrug-resistant organisms. Most of the antibiotics on the WHO reserve list are not available in RS. There is no unified ASP.

In Bosnia and Herzegovina, there is not even a unified reserve antibiotic list. The list of reserve antibiotics of Hospital Saint Apostle Luka (HSAL) consists of cephalosporin second generation (cefuroxime), cephalosporin third generation (ceftazidime, cefotaxime, and ceftriaxone), cephalosporin fourth generation (cefepime), carbapenems (imipenem, meropenem, and ertapenem), glycopeptides (vancomycin and teicoplanin), quinolone (ciprofloxacin, levofloxacin, and moxifloxacin), piperacillin–tazobactam, clarithromycin, colistin, and linezolid. This list is based on literature data, recommendations from other hospitals, which means it is not based on a study of AMR within the hospital.

The aim of this paper is to examine whether the COVID-19 pandemic has influenced the trend of antibiotic consumption in outpatient and inpatient settings in the Doboj Region of the Republic of Srpska (Bosnia and Herzegovina). We compared the consumption of antibiotics in the COVID-19 pandemic era (outpatient antibiotic consumption in 2020; inpatient antibiotic consumption in 2020 and 2021) to the period before the pandemic (2019) and the consumption by selected ATC groups and by AwaRe groups of antibiotics. The second goal was to determine AMR and the presence of multiresistant bacterial strains in hospitalized patients in HSAL in 2021.

## 2 Materials and methods

### 2.1 Design and study population

For this descriptive ecological study, pharmacy-dispensing data on used antibiotics in inpatients settings in HSAL Doboj were obtained from the hospital pharmacy for 2019, 2020, and 2021. The consumption of antibiotics in outpatient settings was calculated based on data from the Public Health Institute (PHI) of Republic of Srpska that provide details of all dispensed antibiotic prescriptions from the community pharmacies in every municipality in the Doboj Region for 2019 and 2020. Doboj is the largest center of this region with more than 200,000 citizens who have access to HSAL. HSAL encompasses 514 beds with approximately 20,000 patients visiting per year.

The consumption of antibiotics in inpatient settings was calculated as the defined daily dose (DDD) per 100 patient-days (PD). PD was calculated as a ratio of the number of inpatient days to the number of patient admissions. The consumption of antibiotics in outpatient settings was calculated as the defined daily dose per 1,000 inhabitants per day (DDD/TID) (total DDD issued in the calendar year in all open-type pharmacies/estimated mid-year population of the Doboj Region 1000/365).

The consumption of antibiotics was calculated according to the specific ATC and AWaRe group (Table 1). According to the ATC group, antimicrobial agents were divided into the following groups: penicillins (J01C), cephalosporins (J01D), macrolides (J01F), quinolone (J01M), tetracyclines (J01A), and other antibiotics including J01G (aminoglycosides), J01FF (lincosamides), and J01XD (imidazole derivatives).

**TABLE 1:** List of antibiotics available in HSAL categorized by the AWaRe classification.

Access	Watch	Reserve
Ampicillin	Azithromycin	Colistin
Amoxicillin	Cefaclor	Fosfomycin
Amoxicillin + clavulanic acid	Cefepime	Linezolid
Benzylpenicillin	Cefixime	
Phenoxymethylpenicillin	Cefopodoxime	
Cloxacillin	Cefotaxime	
Cefalexin	Ceftazidime	
Cefazolin	Ceftriaxone	
Amikacin	Cefuroxime	
Gentamicin	Ciprofloxacin	
Sulfamethoxazole + trimethoprim	Clarithromycin	
Doxycycline	Ertapenem	
Metronidazole	Erythromycin	
Nitrofurantoin	Imipenem + cilastatin	
Clindamycin	Levofloxacin	
Chloramphenicol	Lincomycin	
	Meropenem	
	Moxifloxacin	
	Norfloxacin	
	Piperacillin + tazobactam	
	Roxithromycin	
	Vancomycin	

In the next step, data on isolated bacteria and their sensitivity to antibiotics were collected retrospectively from the protocol of the microbiology department for the year 2021. Resistance of bacteria to antibiotics is expressed as the rate and density for each observed antibiotic. The rate of resistance was calculated as a percentage in relation to the total number of isolates of individual bacteria. The density of resistance of isolated bacteria against a specific antibiotic was expressed as the number of resistant pathogens/1000 patient-days (number per 1000 PD). To examine the rationale for antimicrobial prescribing in hospitals, interviews were conducted with 10 prescribers. All interviewers were physicians, with diverse specialty backgrounds, positions, and number of years in practice. The questionnaire consisted of open-ended questions, allowing the prescribers to expand on their responses or give more details. The interview questions are given in Table 2.

**TABLE 2:** Interview questions.

1. In your opinion, are antimicrobial agents misused or overused in your hospital?
2. What is the main determinant of antimicrobial prescribing in your everyday practice?
3. What hospital services do you consult when prescribing antimicrobial agents?
4. How often do you take samples for culture and susceptibility testing before the start of any treatment with antimicrobial agents?
5. Are you familiar with AMR rates in the hospital or your department?

### 2.2 Statistics

The methods of descriptive statistics were used for data description and analyses. Among the methods of descriptive statistics, numbers and percentages were used. All statistical analyses were performed using IBM SPSS Statistics software version 24.0 for Windows (IBM Corp., Armonk, NY, United States). All *p*-values < 0.05 were considered statistically significant.

## 3 Results

During 2021, 1,095 isolates were taken from a total of 20,918 hospitalized patients with 109,750 patient-days. Of these, the isolates were recovered mostly from urine samples (522), followed by wound swabs (179) and blood cultures (101). According to the type of isolated bacteria, *Escherichia coli* (244) led the way, followed by *S. aureus* (104), *Klebsiella* spp. (97), *Klebsiella pneumoniae* (49), *Proteus* (91), *P. aeruginosa* (62), and *A. baumanii* (44). In 2021, the resistance rate and density of antibiotics in the analyzed samples were as follows: antimicrobial resistance of *A. baumanii* to meropenem was 66% and 0.69 per 1000 PD, gentamicin was 76.3% and 0.79 per 1000 PD, and amikacin was 70.1% and 0.73 per 1000 PD; the most frequent resistance was for ciprofloxacin (91.9% and 0.96 per 1000 PD) and sulfamethoxazole + trimethoprim (91.3% and 0.94 per 1000 PD). Antimicrobial resistance of *Klebsiella* spp. to cefuroxime was 70.2% and 0.90 per 1000 PD, cefotaxim was 67.1% and 0.85 per 1000 PD, cefalexin was 79.3% and 0.70 per 1000 PD; however, the most frequent resistance was for ampicillin (93.8% and 1.25 per 1000 PD) and amoxicillin (91% and 0.84 per 1000 PD). Antimicrobial resistance of *Pseudomonas* to meropenem was 25.7% and 0.24 per 1000 PD and that of *S. aureus* to amoxicillin was 36% and 0.32 per 1000 PD, while coagulase-negative *Staphylococcus* spp*.* were most frequently resistant to ampicillin and amoxicillin (both 79.1% and 0.17 per 1000 PD). *Proteus* was most frequently resistant to amoxicillin (84% and 0.75 per 1000 PD) and ampicillin (82% and 0.84 per 1000 PD), while *E. coli* was most frequently resistant to ampicillin (82% and 1.33 per 1000 PD), amoxicillin (84% and 1.18 per 1000 PD), and amoxicillin + clavulanic acid (76% and 1.17 per 1000 PD) (Tables 3, 4).

**TABLE 3:** Rate of resistance according to the most common multiresistant strains of bacteria at HSAL Doboj in 2021.

J01 antimicrobial agent	R *Acinetobacter*	baumanii I	R *Klebsiella* spp.	I	R *Pseudomonas* spp.	I	R *Staphylococcus*	aureus I	R Coagulase-negative	I *Staphylococcus* spp.	R *Proteus*	I	R *E. coli*	I
Ampicillin	—	—	93.8	6.2	—	—	35.3	40.5	79.1	16.7	82.0	12.6	62.0	37.0
Amoxicillin	—	—	91.0	8.9	—	—	36.0	40.3	79.1	16.7	84.0	15.0	59.1	40.0
Amox/clav	—	—	81.3	6.3	—	—	15.6	35.5	62.5	12.5	76.0	7.00	54.0	23.3
Pip/taz	—	—	24.2	14.1	15.3	38.6	2.3	0.00	36.3	4.6	—	—	—	
Cefalexin	—	—	79.3	6.3	—	—	11.5	7.8	58.3	12.6	66.0	7.0	16.3	23.4
Cefuroxime	—	—	70.2	6.3	—	—	—	—	—	—	67.0	10.0	22.3	9.3
Ceftazidime	—	—	55.2	8.9	26.9	43.3	—	—	—	—	29.0	22.0	12.7	9.4
Ceftriaxone	—	—	38.9	12	—	—	—	—	—	—	26.2	28.1	9.2	9.3
Cefotaxime	—	—	67.1	2.2	—	—	—	—	—	—	59.4	3.9	20.3	4.7
Cefepime	—	—	34.8	17.2	20.4	38.9	—	—	—	—	9.9	7.0	—	—
Imipenem	49.5	10.5	2.2	8.8	24.7	33.8	—	—	—	—	—	—	0.4	0.0
Meropenem	66.0	23.6	8.8	13.9	25.7	11.0	—	9.2		20.9	—	—	1.3	3.5
Erythromycin	—	—				—			33.3		—			—
N			—		—		8.0				—	—	—	—
Clindamycin	—	—		—	—	—	25.0	0.0	75.0	0.0	—	—	—	—
Tetracycline	—	—	—	—	—	—	9.6	27.9	25.0	41.7	—			—
Gentamicin	76.3	15.0	27.8	40.3	16.0	26.0	—	—		—	25.5	40.0	11.8	45.0
Amikacin	70.1	15.9	13.8	23.6	14.8	4.90	6.1	2.5		13.8	20.4	35.0	1.8	28.2
Ciprofloxacin		6.2		6.7		21.7		6.9		22.8	—			7.9
N	91.3		58.8		47.4		27.2					—	30.5	
TMS	91.9	6.2	53.4	10.3	—	—	14.1	15.5		20.9	—	—	37.6	6.4
Colistin	0.0	0.0	—	—	—	—	—	—	—	—	—	—	—	—

R, resistant; S, sensitive; I, intermediate; Amox/clav, amoxicillin/clavulanic acid; Pip/taz, piperacillin/tazobactam; TMS, trimethoprim/sulfamethoxazole.

**TABLE 4:** Resistance density per 1000 patient-days per year according to the most common multiresistant strains of bacteria at HSAL Doboj in 2021.

J01 antimicrobial agent	*A. baumanii*	*Klebsiella* spp.	*Pseudomonas* spp.	*S. aureus*	Coagulase-negative *Staphylococcus* spp.	*Proteus*	*E. coli*
Ampicillin	—	1.25	—	0.32	0.17	0.84	1.33
Amoxicillin	—	0.84	—	—	—	0.75	1.18
Amox/clav	—	1.08	—	0.14	0.14	0.76	1.17
Pip/taz	—	0.30	0.15	—	—	—	—
Cefalexin	—	0.70	—	0.02	0.07	0.45	0.27
Cefuroxime	—	0.90	—	—	—	0.65	0.46
Ceftazidime	—	0.67	0.26	—	—	0.26	0.26
Ceftriaxone	—	0.40	—	—	—	0.25	0.19
Cefotaxime	—	0.85	—	—	—	0.57	0.44
Cefepime	—	0.43	0.19	—	—	0.09	0.09
Imipenem	0.52	0.03	0.23	—	—	—	0.01
Meropenem	0.69	0.11	0.24	—	—	—	0.03
Erythromycin	—	—	—	0.11	0.13	—	—
Clindamycin	—	—	—	0.07	0.07	—	—
Tetracycline	—	—	—	0.02	0.03	—	—
Gentamicin	0.79	0.37	0.07	0.09	0.05	0.26	0.26
Amikacin	0.73	0.16	0.14	0.05	0.04	0.19	0.04
Ciprofloxacin	0.96	—	0.42	0.11	0.10	—	0.61
TMS	0.94	0.71	—	0.09	0.08	—	0.82
Colistin	0	—	—	—	—	—	—

Amox/clav, amoxicillin/clavulanic acid; Pip/taz, piperacillin/tazobactam; TMS, trimethoprim/sulfamethoxazole.

In order to examine the antimicrobial prescribing practice in HSAL, an interview with physicians was conducted. The general characteristics of the interviewed prescribers are given in Table 5. The responses of participants revealed concerns about misuse and overuse of antibiotics, especially in the context of COVID-19. As determinants of antimicrobial prescribing, participants prioritized healthcare system factors (delays in tendering procedures, antimicrobial medicine shortages, and constraint in hospital budgets) and local setting factors (inadequate laboratory services, unclear sensitivity testing forms, lack of locally adapted guidelines for antimicrobial prescribing, and lack of targeted training on antimicrobial stewardship). On the other hand, infection prevention and control were described as factors partially influencing antimicrobial prescribing.

**TABLE 5:** Characteristics of prescribers.

Characteristic	Number of participants, *n* = 10 (%)	
Gender	Male	7 (70)
	Female	3 (30)
Years of practice	5–10	2 (20)
	11–15	3 (30)
	≥15	5 (50)
Position	Head of the department	6 (60)
	Chief resident	2 (20)
	Senior resident	2 (20)
Specialty	Internal medicine	3 (30)
	Infectious diseases	1 (10)
	Obstetrics and gynecology	2 (20)
	Pediatrics	1 (10)
	Surgery	3 (30)

Antibiotic consumption in the hospital setting registered during 2019, 2020, and 2021 was as follows: carbapenems (meropenem: 0.28; 1.91; 2.33 DDD/100 bed-days, respectively).

In their everyday practice, physicians consider themselves independent and often act autonomously in prescribing antimicrobials. They rarely consult other healthcare workers in their decisions. However, prescribers find microbiology services important in guiding antimicrobial prescribing but the disadvantage is the frequent delays in obtaining the results of sensitivity testing. Also, the majority of respondents highlighted unclear hospital sensitivity testing forms, resulting in an uncertainty of the right antibiotic choice. The aforementioned factor was considered a contributing factor for high levels of empirical antibiotic prescribing. None of the respondents was familiar with AMR rates in their hospital or their department.

During 2019, 2020 and 2021 antibiotic consumption in hospital setting was as follows: carbapenems (meropenem: 0.28; 1.91; 2.33 DDD/100 patient-days, respectively), glycopeptides (vancomycin: 0.14; 1.09, 1.54 DDD/100 patient-days, respectively), cephalosporins (ceftriaxone: 6.69; 14.7; 14.0 DDD/100 patient-days, respectively) and polymyxins (colistin: 0.04; 0.25; 0.35 DDD/100 patient-days, respectively). The consumption of azithromycin increased drastically in 2020 and dropped significantly in 2021 (0.48; 5.61; 0.93 DDD/100 patient-days) (Table 6).

**TABLE 6:** Defined daily dose per 100 patient-days of antibiotics before (2019) and during the pandemic (2020 and 2021) in HSAL Doboj per year.

Groups of antibiotics	INN of reserved antibiotics	ATC	Antibiotic use at HSAL Doboj (DDD/100 patient-days)		

			2019	2020	2021
Glycopeptides	Vancomycin	J01XA01	0.14	1.09	1.54
Aminoglycosides	Amikacin	J01GB06	1.13	2.11	1.75
Carbapenems	Imipenem/cilastatin	J01DH51	0.29	0.93	1.22
	Meropenem	J01DH02	0.28	1.91	2.33
Beta-lactam/beta-lactamase inhibitor	Piperacillin/tazobactam	J01CR05	0.02	0.06	0.09
Oxazolidines	Linezolid	J01XX08	0.00	0.00	0.02
Cephalosporins	Ceftriaxone	J01DD04	6.69	14.70	13.98
	Cefepime	J01DE01	0.06	0.16	0.21
	Cefotaxime	J01DD01	0.00	0.00	0.00
	Ceftazidime	J01DD02	0.02	0.00	0.00
Polymyxins	Colistin	J01XB01	0.04	0.25	0.35
Macrolides	Azithromycin	J01FA10	0.48	5.61	0.93

INN, International Non-proprietary Name.

The total consumption of antibiotics for systemic use (J01) in outpatient settings in the Doboj Region in the Republic of Srpska was not changed in the observed period of time, with 13.75 DDD/TID in 2019 and 13.55 DDD/TID in 2020. A higher use of cephalosporins (from 1.38 to 1.63 DDD/TID) and macrolides (from 1.07 to 1.42 DDD/TID) was observed during the pandemic period than the period before the COVID-19 pandemic (Table 7). The ratio of macrolide use between 2019 and 2020 was 1.32, while for cephalosporins, it was 1.18. The consumption of penicillins, quinolones, and other antibiotics of the J01 group did not change significantly in the studied period (DDD/TID 2020 and DDD/TID 2019 ratios were 0.89, 0.98, and 1.11, respectively). In the outpatient setting, an increase in the consumption of oral forms of azithromycin, levofloxacin, and cefixime and parenteral forms of co-amoxiclav, ciprofloxacin, and ceftriaxone was recorded (Table 7).

**TABLE 7:** Defined daily dose per 1,000 inhabitants per day of antibiotics for systemic in the outpatient setting before (2019) and during the pandemic period (2020) in HSAL Doboj Region per year.

Groups of antibiotics	INN	ATC	Antibiotic use at HSAL Doboj (DDD/100 patient-days)		Relative rate of change*

			2019	2020	
Penicillins	Phenoxymethylpenicillin	J01CEO2	0.53	0.41	0.77
	Amoxicillin	J01CA04	4.49	3.91	0.87
	Ampicillin po	J01CA01	0.63	0.18	0.29
	Ampicillin pe		0.05	0.05	1.09
	Amox/clav po	J01CR02	1.50	1.82	1.22
	Amox/clav pe		0.00	0.01	2.57
Total			7.19	6.38	0.89
Cephalosporins	Cefalexin	JO1DB01	0.77	0.89	1.17
SUM first generation			0.77	0.89	1.17
	Cefuroxime po	J01DC02	0.36	0.37	1.03
	Cefuroxime pe		0.00	0.00	0.63
	Cefaclor	J01DC04	0.02	0.01	0.52
SUM second generation			0.38	0.38	1.00
	Cefixime po	J01DD08	0.09	0.15	1.62
	Cefpodoxime	J01DD13	0.03	0.03	1.01
	Ceftriaxone	J01DD04	0.11	0.18	1.60
SUM third generation			0.23	0.36	1.54
Total			1.38	1.63	1.18
Macrolides	Azithromycin po	J01FA10	0.66	1.18	1.77
	Azithromycin pe		0.00	0.00	—
	Erythromycin	J01FA01	0.19	0.06	0.32
	Clarithromycin po	J01FA09	0.22	0.18	0.81
	Clarithromycin pe		0.00	0.00	—
	Roxithromycin	J01FA06	0.00	0.00	0.35
Total			1.07	1.42	1.32
Quinolones	Ciprofloxacin po	J01MA02	0.46	0.41	0.90
	Ciprofloxacin pe		0.00	0.01	1.65
	Levofloxacin	J01MA12	0.14	0.24	1.74
	Moxifloxacin po	J01MA14	0.01	0.01	0.94
	Moxifloxacin pe		0.00	0.00	6.65
	Norfloxacin	J01MA06	0.46	0.37	0.81
Total			1.07	1.05	0.98
Others	Doxycycline	J01AA02	0.64	0.41	0.63
	TMS	J01EE01	0.54	0.27	0.50
	Lincomycin po	J01FF02	0.10	0.06	0.56
	Lincomycin pe		0.00	0.00	1.26
	Clindamycin po	J01FF01	0.04	0.04	1.05
	Clindamycin pe		0.00	0.00	4.67
	Gentamicin	J01GB03	0.68	0.92	1.34
	Nitrofurantoin	J01XE01	0.12	0.23	1.74
	Metronidazole po	J01XD01	0.91	1.05	1.16
	Metronidazole pe		0.01	0.12	0.11
Total			2.40	2.67	1.11
Total	All INN				

DDD/TID—defined daily dose per 1,000 inhabitants per day; INN, International Non-proprietary Name. *relative rate of change is calculated (DDD/TID 2020/DDD/TID 2019); po, per oral; pe parenteral.

## 4 Discussion

Despite COVID-19 being a viral disease, antibiotic treatment including broad-spectrum antibiotics was prescribed to all patients regardless of the severity of illness, especially at the beginning of the pandemic. This practice was supported by reported, suspected, or confirmed secondary bacterial infections in COVID-19 patients (Khan et al., 2022). High consumption of antibiotics was already observed in April 2020, which raised concerns that the misuse or overuse of antibiotics could contribute to the development and spread of antimicrobial resistance. Therefore, the introduction of clear clinical guidelines led to the decrease in antibiotic consumption later in 2020 (Ghosh et al., 2021; Roche et al., 2022). Our study revealed that the overall consumption of antibiotics in the Doboj Region did not significantly change during the pandemic, when compared to the pre-pandemic year. However, an increase in the consumption of amoxicillin, azithromycin, cephalexin, gentamicin, ciprofloxacin, cefuroxime, and sulfamethoxazole + trimethoprim was observed in outpatient settings, while an increase in the consumption of ceftriaxone, meropenem, vancomycin, colistin, and azithromycin was observed in inpatient settings. The year 2020 was marked by an enormous increase in the consumption of azithromycin both in outpatient and inpatient settings. These findings are similar to those reported from the surrounding areas and countries around the world (ESAC-Net, 2022; Sokolović et al., 2022). An increase in the consumption of azithromycin could be partly explained due to its immunomodulatory and antiviral properties, in addition to its antibacterial effects (Sultana et al., 2020).

Many clinical studies have shown that the use of azithromycin did not have any positive effect in the treatment of patients with COVID-19 infection (Del Fiol et al., 2022). In the newer version of the guidelines for the treatment of patients with COVID-19, strong recommendations were made against the use of azithromycin alone and azithromycin in combination with hydroxychloroquine and colchicine (Bogdanić et al., 2022; Del Fiol et al., 2022; Roche et al., 2022). Regarding inpatient settings, the hospital list of restricted antibiotics that require an approval from the designated infection control team when used consists of 15 antibiotics (piperacillin/tazobactam, cefuroxime, ceftazidime, ceftriaxone, cefotaxime, cefepime, imipenem/cilastatin, meropenem, ertrapenem, vancomycin, colistin, clarithromycin, moxifloxacin, ciprofloxacin, and levofloxacin). The antibiotics listed are not classified under the Watch or Reserve group according to the AWaRe classification (World Health Organization, 2019a). Surprisingly, azithromycin is not included in the list, even though it is included in the Watch antibiotic group in the AWaRe classification. Requiring no preauthorization for azithromycin use potentially led to its misuse and high consumption in 2020. Also, linezolid is not included in the list, although it is included in the Reserve antibiotic group in the AWaRe classification; however, this fact did not change its consumption in 2020. However, a slight increase in linezolid consumption in 2021 is an important factor for the development and spread of AMR (Grau et al., 2021b). Other antibiotics included in the list of restricted antibiotics (imipenem/cilastatin, meropenem, and vancomycin) had markedly increased consumptions in 2020 when compared to that in 2019, with a further increase in 2021, with the exception of ceftriaxone, whose consumption slightly decreased in 2021. This suggests that preauthorization for restricted antibiotic use is either not strictly adhered to or feedback interventions on antibiotic prescribing (review of antibiotic prescribing and dose optimization) are lacking or both (World Health Organization, 2019b). The aforementioned studies point out that the hospital list of restricted antibiotics needs to be improved in order to better support antibiotic monitoring and antibiotic stewardship activities.

Compared with the European Surveillance of Antimicrobial Consumption Network (ESAC-Net) (ESAC-Net, 2022), we concluded that consumption in the Doboj Region in 2020 is similar to the level of Croatia (14 DDD/TID), Slovakia (13.2 DDD/TID), Malta (14.4 DDD/TID), Denmark (12.5 DDD/TID), and Luxembourg (14.8 DDD/TID), and individually according to ATC groups, they are most similar to Croatia and Luxembourg. Considering the change in total consumption, consumption was maintained at the same level only in Denmark (13.4 DDD/TID), while Croatia, Slovakia, Luxembourg, and Malta showed a reduction in consumption in 2020 compared to that in 2019 (DDD/TID in 2019: 16.9; 18.0; 19.8; 18.7, respectively).

In most European countries, ASP has already been introduced and is strictly applied, which has already led to a decrease in the consumption of antibiotics in outpatient and hospital settings. The result is that in recent years, a stable level of resistance to antibiotics has been registered for most bacteria that cause infections in humans, with the exception of *Enterococcus faecium*. The resistance of this bacterium to vancomycin increased from 9% in 2014 to 17% in 2020. The resistance of *P. aeruginosa* to carbapenems in European countries in 2020 was estimated to be 20%. The results of antimicrobial resistance to restricted antibiotics (e.g., meropenem, ciprofloxacin, and ceftriaxone) showed high rates for the most common multiresistant strains of bacteria at HSAL Doboj. The resistance of *P. aeruginosa* to meropenem was 25.7%, which is a marker that warns of a high degree of resistance. *A. baumannii* is an opportunistic pathogen with the ability to survive in hospital environments for a long time and gain many virulence factors, emerging as an important nosocomial pathogen. Several factors could have contributed to the increased isolation frequency of this pathogen during the pandemic: the increased clinical severity of hospitalized cases, the increased duration of hospitalization, and the increased use of antibiotics mainly carbapenems, mostly in the ICU setting (Polemis et al., 2021). The resistance of *Acinetobacter* spp. to carbapenems in HSAL Doboj was 66.6%, which greatly exceeds the resistance of this bacterium in European countries in 2020, which was 38%. In 2021, the trend of increasing consumption of reserve antibiotics in hospital conditions continued (European Centre for Disease Prevention and Control, 2022).

This study has some strengths: the size of the studied sample, distinct rural and urban environments, and the incorporation of a diverse population that included intensive care unit, psychiatric, internal medicine, and pediatric participants, enabling us to draw conclusions that could be applied to diverse population. It is also relevant to bear in mind that the data were collected for 3 years, over the pandemic, and may not reflect the current use or resistance issues. Therefore, extrapolating these results to different periods of time could be misleading. Future studies are on their way to investigate other regional hospitals over a longer period of time, allowing us to inquire about temporal patterns.

This study had several limitations. First, we only collected antibiotic prescribing data in one regional hospital that covers a large population of patients, but we cannot be certain that the prescribing practices observed were representative of other hospitals in Bosnia and Herzegovina. Second, we did not measure the severity and clinical course of infection, the duration of antibiotic use, or whether clinical staff altered the route of antibiotic administration over time or if these prescription patterns were based on the microbiology test result after the hospital had been surveyed. Third, we could not completely assess national- or hospital-specific guidelines due to their unavailability for all encountered diagnoses. Additionally, some guidelines that recommended antimicrobial use did not specify which antimicrobials should be used. Fourth, we did not assess guideline compliance in relation to antimicrobial dose, timing of administration, or appropriateness of therapy decisions. Finally, the prevalence of the identified pathogen-directed antimicrobial prescription was likely underestimated due to limited healthcare resources, including laboratory services in the hospital. This led to the conclusion that a significant prevalence of appropriate indication for empirical therapy could be overestimated. Our main results underscore the need to update evidence-based guidelines for antibiotic use, promote the benefits of targeted therapy, and ensure the implementation of hospital-based antimicrobial stewardship programs at the hospitals surveyed. This kind of analysis makes it possible to propose an adequate ASP for the mentioned hospital.

It is necessary to introduce clear recommendations for the use of antimicrobial agents, to sample them more frequently, and to make empirical treatment less prevalent. Also, the precise guidelines for the empirical use of antibiotics should be adopted with appropriately educating physicians. The given results of antibiotic resistance in HSAL should be implemented in a new list of reserve antibiotics. The method of taking antibiotics from the hospital pharmacy to hospital wards should be more precisely defined and oriented to the specific patient. The introduction of stewardship programs in hospitals could reduce the consumption of antibiotics, which would have a positive impact on the reduction of antimicrobial resistance.

## Data Availability

The raw data supporting the conclusion of this article will be made available by the authors, without undue reservation.
